# Expression of RHOGTPase regulators in human myometrium

**DOI:** 10.1186/1477-7827-6-1

**Published:** 2008-01-11

**Authors:** Margaret O'Brien, David Flynn, Brian Mullins, John J Morrison, Terry J Smith

**Affiliations:** 1 National Centre for Biomedical and Engineering Science, Orbsen Building, National University of Ireland Galway, University Road, Galway, Ireland; 2 Department of Obstetrics and Gynaecology, National University of Ireland Galway, Clinical Science Institute, University College Hospital Galway, Newcastle Road, Galway, Ireland

## Abstract

**Background:**

RHOGTPases play a significant role in modulating myometrial contractility in uterine smooth muscle. They are regulated by at least three families of proteins, RHO guanine nucleotide exchange factors (RHOGEFs), RHOGTPase-activating proteins (RHOGAPs) and RHO guanine nucleotide inhibitors (RHOGDIs). RHOGEFs activate RHOGTPases from the inactive GDP-bound to the active GTP-bound form. RHOGAPs deactivate RHOGTPases by accelerating the intrinsic GTPase activity of the RHOGTPases, converting them from the active to the inactive form. RHOGDIs bind to GDP-bound RHOGTPases and sequester them in the cytosol, thereby inhibiting their activity. Ezrin-Radixin-Moesin (ERM) proteins regulate the cortical actin cytoskeleton, and an ERM protein, moesin (MSN), is activated by and can also activate RHOGTPases.

**Methods:**

We therefore investigated the expression of various RHOGEFs, RHOGAPs, a RHOGDI and MSN in human myometrium, by semi-quantitative reverse transcription PCR, real-time fluorescence RT-PCR, western blotting and immunofluorescence microscopy. Expression of these molecules was also examined in myometrial smooth muscle cells.

**Results:**

ARHGEF1, ARHGEF11, ARHGEF12, ARHGAP5, ARHGAP24, ARHGDIA and MSN mRNA and protein expression was confirmed in human myometrium at term pregnancy, at labour and in the non-pregnant state. Furthermore, their expression was detected in myometrial smooth muscle cells. It was determined that ARHGAP24 mRNA expression significantly increased at labour in comparison to the non-labour state.

**Conclusion:**

This study demonstrated for the first time the expression of the RHOGTPase regulators ARHGEF1, ARHGEF11, ARHGEF12, ARHGAP5, ARHGAP24, ARHGDIA and MSN in human myometrium, at term pregnancy, at labour, in the non-pregnant state and also in myometrial smooth muscle cells. ARHGAP24 mRNA expression significantly increased at labour in comparison to the non-labouring state. Further investigation of these molecules may enable us to further our knowledge of RHOGTPase regulation in human myometrium during pregnancy and labour.

## Background

Myometrial smooth muscle contractility is regulated by both intracellular calcium concentration ([Ca^2+^]_i_) and by the calcium sensitivity of myofilaments [1]. Calcium sensitisation refers to an increase in smooth muscle tension and/or phosphorylation of MLC at a constant [Ca^2+^]_i _by inhibition of myosin light chain phosphatase (MLCP). RHOA, a member of the RHOGTPase subfamily, the RAS family of G proteins, is the upstream component along with two target proteins, RHO-associated coil-forming kinase (ROCKI) and its isoform, ROCK2, which are associated with inhibition of MLCP [2]. The RHO family of small GTPases consists of at least 20 members which includes RHO, RAC, CDC42 and RND [3]. *RHOA *mRNA expression increased in human labouring myometrium while active RHOA levels increased in pregnancy and spontaneous preterm labour myometrium [4,5]. *RHOB *mRNA and RND2 and 3 protein expression increased during pregnancy while RHOD protein levels decreased at labour, in comparison to the non-pregnant state [5,6]. RHOGTPases are highly regulated, they cycle between an active GTP-bound and an inactive GDP-bound state and this cycling is regulated by at least three distinct protein families: RHOGTPase guanine nucleotide exchange factors (GEFs), RHO activating proteins (GAPs) and RHO guanine nucleotide dissociation inhibitors (GDIs) [7-9].

RHOGEFs activate RHOGTPases by catalysing GTP for GDP exchange. They contain a Dbl homology domain which is responsible for the catalysis of GDP to GTP and a pleckstrin homology domain which mediates membrane localisation [10]. Some 70 RHOGEFs have been identified in the human geneome [10]. ARHGEF1, (also known as p115-RHOGEF), ARHGEF11 (PDZ-RHOGEF) and ARHGEF12 (LARG) are members of a subgroup of RHOGEFs, known as regulators of G protein signalling (RGS) domain containing RHOGEFs. The RGS domain mediates their binding to and activation of Gα_12/13 _(and Gq also in the case of ARHGEF12), in response to G-protein coupled receptor activation [11,12]. All three exhibit very selective activation of RHOA [13]. Moreover, ARHGEF11 induces calcium sensitisation in the absence of agonist mediated signalling in rabbit pulmonary artery smooth muscle [14], its expression being up-regulated by estradiol treatment in guinea pig myometrium [15].

RHOGAPs accelerate the low intrinsic GTP hydrolysis rate of most RHO family members, converting the proteins to the GDP-bound inactive conformation [16]. RHOGAPs share a related 140 amino acid domain, that interacts with the GTP-binding core of RHOGTPases [8]. Over 70 RHOGAPs have been identified in eucaryotes [17]. ARHGAP24 (also known as p73-RHOGAP, FilGAP or RC-GAP72) is a filamin A-binding RHOGTPase-activating protein involved in cell polarity, cell morphology and cytoskeletal organisation and is also a key angiogenic regulator [18-20]. ARHGAP24 acts a specific GTPase activator for RAC, it controls actin remodelling by inactivating RAC, downstream of Rho, which suppresses leading edge protrusion and promotes cell retraction to achieve cell polarity [19]. ARHGAP5 (p190B-RHOGAP) is ubiquitously expressed and has *in vitro *specificity for RHOA, RAC and CDC42 [8]. Inhibition of contractility in rat vascular SMCs is associated with recruitment and accumulation of its isoform, p190-RHOGAP, and its subsequent inactivation of RHOA [21,22].

RHOGDIs bind to RHOGTPases, which are prenylated, and sequester them in the cytosol, thereby inhibiting their activity [9]. There are three mammalian RHO family-specific GDIs, ARHGDIA (also known as RHOGDIα, RHOGDI or RHOGDI 1), RHOGDIβ and RHOGDIγ. ARHGDIA binds to RHOA and B, RAC1 and 2, and CDC42 both *in vitro *and *in vivo*. In addition to their role in the inhibition of RHOGTPases RHOGDIs may also act as positive regulators for the targetting and regulation of RHO activities [23].

Moesin (membrane-organizing extension spike protein, MSN) is an ERM (Ezrin-Radixin-Moesin) regulatory protein and member of the 4.1 super-family [24]. ERM proteins cross-link cortical actin filaments with the plasma membrane and are involved in the formation of microvilli, cell-cell adhesion, maintenance of cell shape and motility, and membrane trafficking [25]. MSN activation is achieved through phosphorylation on Thr^558 ^by ROCK2 [26]. Modulation of ERM protein activity can also contribute to GTPase activation, the conserved N-terminal FERM domain of ERM proteins can bind to RHOGDI and displace it from RHOA, RAC1 and CDC42 [27]. Furthermore, it was observed that interaction of MSN with RHOGDI resulted in RHOA activation [28].

As the role of RHOGTPases in myometrial contractility continues to be elucidated, regulation of these important proteins is essential, particularly during labour. Our study aimed to investigate the expression of ARHGEF1, ARHGEF11, ARHGEF12, ARHGAP5, ARHGAP24, ARHGDIA and MSN in the human myometrium utilising RT-PCR, western blotting and immunofluorescence techniques.

## Methods

### Tissue collection

Biopsies of human myometrial tissue during pregnancy were obtained at elective (pregnant not in labour, PNL) and intrapartum (pregnant in labour, PL) and preterm emergency caesarean delivery (prior to 34 weeks, PT). The biopsies were excised from the upper lip of the lower uterine segment incision in the midline, i.e. the upper portion of the lower uterine segment. Biopsies of human non-pregnant myometrial tissue (non-pregnant, NP) were obtained from pre-menopausal hysterectomy specimens and women receiving therapy with progestagens, luteinising hormone-releasing hormone analogues, or steroid based medications were excluded from the study. Women who had received prostaglandins or oxytocin for either induction or augmentation of labor were excluded from the study. Women with gynaecologic malignancy were also excluded from the study. Myometrial samples were carefully dissected to minimize decidual inclusion. Ethical Committee approval for the tissue collection procedure was obtained from the Research Ethics Committee University College Hospital, Galway and recruitment was by written informed consent. Immediately upon collection, tissue was rinsed in normal saline for myometrial cell isolation or snap-frozen in liquid nitrogen, and stored at -80°C.

### Tissue samples for semi-quantitative RT-PCR

Myometrial biopsies were obtained at hysterectomy (*n *= 1), elective (*n *= 2) and intrapartum caesarean section (*n *= 2). The reason for hysterectomy was menorrhagia, aged 45. The reasons for elective caesarean section included previous caesarean section (*n *= 2). The reasons for emergency caesarean section were face presentation (*n *= 2). The mean age of the caesarean section women was 38.5 years (range, 36–41) and all the caesarean sections were multigravida and delivered between 37 and 39 weeks' gestation.

### Tissue samples for real-time fluorescence RT-PCR

For real-time RT-PCR confirmation analysis, biopsies of myometrium were obtained at the time of elective (*n *= 7) and intrapartum (*n *= 7) caesarean section. The reasons for elective caesarean section included previous caesarean section (*n *= 6) and placenta praevia (*n *= 1). The reasons for emergency caesarean section were face presentation (*n *= 4), non-reassuring foetal testing (*n *= 1) and previous classical caesarean section (*n *= 2). The mean age of each group was PNL, 34.4 and PL, 34.3 and the overall mean age was 34.35 years (range, 29–41), 3 of whom were primagravida and 11 multigravida. All women were delivered between 37 and 41 weeks' gestation.

### Tissue samples for protein expression

Biopsies of myometrium (for the RHOGEF GAP and GDI tissue westerns) during pregnancy were obtained at elective (*n *= 4) and intrapartum (*n *= 4) caesarean section and at hysterectomy (*n *= 4). The reasons for elective caesarean section were previous caesarean sections (*n *= 4). The reasons for emergency caesarean delivery were non-reassuring foetal testing (*n *= 2), face presentation (*n *= 1) and previous classical caesarean section (*n *= 1). The mean age of the women was 33 years (range 31–37), 4 were primagravida and 4 were multigravida. All women were delivered between 39 and 40 weeks' gestation. The hysterectomies were pre-menopausal (*n *= 4) with a mean age of 46.

Biopsies of myometrium during pregnancy for the first moesin western blots, were obtained at elective (*n *= 3) and intrapartum (*n *= 3) caesarean section. The reasons for elective caesarean section included maternal request (*n *= 1) and previous caesarean section (*n *= 2). The reasons for emergency caesarean delivery were non-reassuring foetal testing (*n *= 1), failed induction (*n *= 1) and failure to progress (*n *= 1). The mean age of the women was 35.5 years (range 30–41), 3 were primagravida and 3 were multigravida. All women were delivered between 39 and 40 weeks' gestation.

Biopsies of myometrium for the last set of moesin western blots, during pregnancy were obtained at intrapartum (*n *= 3) and preterm (*n *= 3) caesarean sections. The reasons for emergency caesarean delivery were non-reassuring foetal testing (*n *= 2) and undiagnosed breech birth (*n *= 1). The reasons for preterm birth were HELLP syndrome (*n *= 1), foetal abnormality (*n *= 1) and ulcerative colitis (*n *= 1). The mean age of the women was 35.5 years (range, 29–37) of which 2 were primagravida and 2 were multigravida. The preterm women were delivered at 32 weeks gestation. The elective and emergency caesarean sections were delivered between 39 and 41 weeks gestation.

### Myometrial cell isolation and cell culture

Myometrial tissue samples were minced (finely with any fibrous tissue removed) and digested in sterile filtered Dulbecco's Modified Eagle Medium (DMEM) (minus calf serum) (Sigma-Aldrich, Ireland) containing collagenase type IA (Sigma-Aldrich, Ireland), collagenase type XI (Sigma-Aldrich, Ireland) and 0.1% bovine serum albumin (w/v) (BSA) (Sigma-Aldrich, Ireland) for 45 minutes. The resulting suspension was vortexed and the non-dispersed tissue fragments were separated by filtration of the mixture through sterile gauze layers. Individual cells were then collected by centrifugation at 400 g for 10 minutes. Cells were then washed and centrifuged 2 to 3 times in sterile 1X phosphate-buffered saline (PBS). After washing, cells were cultured in SGM-2 medium (Cambrex, Biowhittaker UK Ltd., Berkshire, UK) at 37°C and 5% CO_2_. Cells were sub-cultivated with trypsin/ethylenediaminetetraacetic acid (EDTA) at a 1:2 or 1:3 split after reaching confluence. Myometrial cells used in experiments were used to passage 8.

Myometrial smooth muscle cells were characterised for mRNA expression of calponin, and estrogen receptor α and for SM α-actin mRNA and protein expression.

### RNA extraction and reverse transcription

Total RNA was isolated from myometrial tissue using TRIzol reagent (Life Technologies Ltd., UK) [29] and from myometrial smooth muscle cells using the RNeasy mini RNA isolation kit (Qiagen, Crawley, West Sussex, UK). All RNA samples were DNase I treated using the DNA-*free*™ kit (Ambion, Spitfire Close, Huntingdon, Cambridgeshire, UK). RNA (500 ng – DNase I treated) was reverse transcribed into complementary DNA (cDNA) for use as a template for Polymerase Chain Reaction (PCR). The RNA samples were then denatured at 65°C for 10 minutes. Reverse transcription was performed at 42°C for 60 minutes in a reaction volume of 20 μl containing the following: oligo dT primer (500 ng), Moloney murine leukaemia virus (M-MLV) reverse transcription buffer (50 mol l^-3 ^Tris-HCl, pH 8.3, 75 mol l^-3 ^KCl, 3 mol l^-3 ^MgCl_2_, 10 mol l^-3 ^dithiothreitol (DTT)) (Promega, Southampton Science Park, Southampton, UK), diethylpyrocarbonate (DEPC) treated water (BDH, UK), deoxyribonucleotide triphosphates (dNTPs) (0.2 mol l^-3^) (Promega, UK) and 200U M-MLV reverse transcriptase (Promega, UK). Reverse transcriptase activity was stopped by heating samples at 65°C for 10 minutes. Control RNA samples, in which no reverse transcriptase was added, were included to confirm that no genomic DNA contamination was present.

### Semi-quantitative PCR

0.5–1 μl of this 20 μl reaction was then used in the subsequent PCR. The PCR reaction was performed in a final volume of 50 μl containing 1.5 mol l^-3 ^MgCl_2_, 20 mol l^-3 ^Tris-HCl, 50 mol l^-3^, KCl pH 8.3 (Promega, UK), 1.25 U Taq DNA polymerase (Promega., UK), 40 mol l^-6 ^dNTPs and 10 mol l^-12 ^of each sense and antisense primer. cDNA amplification was carried out by an initial denaturation step of 5 minutes at 95°C followed by 34 cycles of denaturation at 94°C for 20 s, annealing at 57°C for 45 s and elongation at 72°C for 45 s, followed by a final extension step at 72°C for 10 minutes. 10 μl of each PCR product was then separated by gel electrophoresis on 1–1.5% (w/v) agarose gels. Products were separated alongside a 100 bp DNA molecular weight ladder (Promega, UK) for sizing. The oligonucleotides synthesised (MWG, Ebersberg, Germany) to PCR amplify the genes of interest were:

*ARHGEF1 *Sense 5'-AGCGAGTTCAAGAACCTGGA-3'

Antisense 5'-TCGTATATCTGGGCCTCCTG-3' [Genbank: NM_198977]

*ARHGEF11 *Sense 5'-GTCTCGAAAGGCAGAGAACG-3'

Antisense 5'-ATCCTTGCCCACTGTATGCT-3' [Genbank: NM_198236]

*ARHGEF12 *Sense 5'-GGAGCATCTGGGAATATGGA-3'

Antisense 5'-TCTTGCAGCTGAGGAATGTG-3' [Genbank: NM_015313]

*ARHGAP5 *Sense 5'-TCCCCCATCCTATACCATCA-3'

Antisense 5'-CAGGCATTTGCTTCTGTTCA-3' [Genbank: NM_001173]

*ARHGAP24 *Sense 5'-GGGCAACAGCAGCAACCACA-3'

Antisense 5'-TCGCTCGGCATTTCGCATTTTTAT-3' [18]

*ARHGDIA *Sense 5'-CATCCAGATCCAGGAGC-3'

Antisense 5'-GACTTGATGCTGTAGCTGCC-3' [30]

*MSN *Sense 5'-CTGATGGAGAGGCTGAAGCA-3'

Antisense 5'-TCTTGGACTCATCTCTGGCA-3' [31]

*ACTB *Sense: 5'-CAACTCCATCATGAAGTGTGAC-3',

Antisense 5'-GCCATGCCAATCTCTCATCTTG-3' [Genbank: M10277]

### Real-time fluorescence PCR using ABI Prism 7000 technology

Real-time PCR was performed on a 1/125 dilution of each the 7 PNL and 7 PL myometrial cDNA in triplicate for each transcript, using the Applied Biosystems ABI Prism 7000 sequence Detection System (ABI, USA). The PCR reactions were performed in a final volume of 25 μl containing 12.5 μl Sybr Green PCR Master Mix (ABI, USA), 5 μl diluted cDNA and 0.4 μM of each sense and antisense primer. The final volume of 25 μl was achieved using PCR grade water (Sigma-Aldrich, Ireland). cDNA amplification was performed by an initial step of 50°C for 2 minutes an initial denaturation step at 95°C for 10 minutes, followed by 40 cycles of denaturation at 95°C for 15 seconds, annealing at 60°C and elongation at 72°C for 30 seconds each. The sequences of the real time fluorescence RT-PCR oligonucleotide primers for the genes are as above, except *ARHGEF12 *and *ARHGDIA*, as alternative primers for these genes were utilised for the real time assay:

*ARHGEF12 *Sense 5'-TGTTGGTGACTCTCGGTTCA-3'

Antisense 5'-CCAATGAGTGGCACACAGTCT-3' [32]

*ARHGDIA *Sense 5'-CAGGAAAGGCGTCAAGATTG-3'

Antisense 5'-GTCAGGAACTCGTACTCCTC-3' [33]

Fluorescence data was acquired at the end of each PCR cycle. Melting curve analysis was performed by an initial denaturation step of 95°C for 15 seconds, cooling to 60°C for 10 seconds, and 72°C for 15 seconds. Fluorescence was measured continually during the melting curve cycle. The mean Cycle Threshold (Ct) of each gene for every patient (performed in triplicate) from their standard curves was normalised to the corresponding mean Ct value of β-Actin (ACTB), *Gene *Ct values/*ACTB *Ct values). β-Actin is a housekeeping gene, it is constitutively expressed and is used to normalise mRNA levels between different samples. The normalised Ct values of the 7 PL and the 7 PNL myometrial tissue types (PL v PNL) were analysed using the independent samples t test. Results were expressed as mean normalised Ct units ± the standard error of the mean (SEM). A P value of < 0.05 was considered to be statistically significant. Relative fold changes were then calculated using the difference in the mean normalised Ct values (x) between the pregnant at-term and the labouring myometrium for each transcript, Relative fold change = 2^x^. All statistical analysis was performed using the SPSS statistical package using one way ANOVA with Tukey's post hoc analysis (Statistical Package for the Social Sciences, v.11, SPSS Inc., Chicago, IL, USA) and GraphPad Prism version 4 (GraphPad Software Inc., San Diego, CA, USA). Real time fluorescence RT-PCR products were gel electrophoresed, bands extracted, purified with the Qiaquick Gel Extraction kit (Qiagen, UK) and DNA sequence verified (MWG, Germany).

### Protein isolation

Human myometrial tissue was homogenised in ice-cold buffer containing 1% Triton-X (Sigma-Aldrich, Dublin, Ireland) and 0.1% sodium dodecyl sulphate (w/v) (SDS) (Sigma-Aldrich, Ireland). Cellular debris was removed by centrifugation at 10,000 × *g*, 4C for 15 minutes. The resultant supernatant was used for Western blot analysis. Human myometrial tissue or myometrial smooth muscle cells or Hela cells were homogenised in Protein lysis buffer: 50 mM Tris pH 7.4, 100 mM NaCl, 5 mM MgCl2, 0.1% Triton X-100, 10% glycerol with inhibitors (10 μg/ml leupeptin, 10 μg/ml aprotinin, 1 mM PMSF) ice-cold buffer (Sigma-Aldrich, Ireland). Cellular debris was removed by centrifugation at 10,000 × *g*, 4°C for 15 minutes. The resultant supernatant was used for Western blot analysis. Protein concentrations were determined using the BCA protein assay reagent kit (Pierce Technology, Rockford, Illinois, USA) as per the manufacturer's protocol, with bovine serum albumin as a standard.

### Western blot analysis

Protein samples (30 μg) were heated at 95°C for 5 minutes after addition of an equal volume of 2X sample buffer containing: 62.5 mol l^-3 ^Tris pH 6.8, 2% SDS (w/v) (Sigma-Aldrich, Ireland), 10% glycerol (v/v) (BDH, Alkem Chemical Ltd, Dublin, Ireland), 5% 2-β mercaptoethanol w/v (Sigma-Aldrich, Ireland) or 5X Laemmli containing 50 mM DTT. This preparation was resolved by electrophoresis on 4–20% (Pierce Technology, USA) or 12% SDS (w/v) polyacrylamide gel electrophoresis (SDS-PAGE) gel at 100 V, room temperature for 60 minutes (BioRad, Hercules, CA, USA) in a buffer containing 25 mol l^-3 ^Tris base, pH 8.3, 19.2 mol l^-2 ^glycine, and 0.1% (w/v) SDS. The separated proteins were transferred to 0.2 μm nitrocellulose or PVDF membranes at a constant voltage of 100 V at 4C for 60 minutes in transfer buffer containing 25 mol l^-3 ^Tris base, 192 mol l^-3 ^glycine, and 20% methanol (v/v), high-performance liquid chromatography (HPLC) grade (Sigma-Aldrich, Ireland). To ensure transfer and equal loading of proteins, blots were stained with Ponceau S solution (Sigma-Aldrich, Ireland) for 5 minutes, followed by washing with de-ionised water, rapid immersion with agitation in 0.1 mol l sodium hydroxide (Sigma-Aldrich, Ireland), and finally washing under flowing de-ionised water for 3 minutes. Membranes were incubated for 1 hour at room temperature with phosphate-buffered saline (PBS; 1 mol l^-2 ^phosphate buffer, 2.7 mol l^-3 ^potassium chloride, and 1.37 mol l^-1 ^sodium chloride, pH 7.4) containing 0.05% Tween 20 (v/v) (Sigma-Aldrich, Ireland) and 5% low-fat milk powder (w/v) (Dawn Dairies, Westmeath, Ireland) to block nonspecific binding. Blots were either incubated for 60 minutes at room temperature or overnight at 4°C, with 1:200 dilution FilGAP antibody or a 1:100 dilution of p115RhoGEF (H-165) sc-20804, 1:100 dilution LARG (H-70) sc-25638, 1:100 dilution p190B RhoGAP (H-160) sc-30206) or 1:750–1000 dilution of RHOGDI (ARHGDIA) (A20 sc360 Santa Cruz Biotechnology, Inc, Heidelberg, Germany) rabbit polyclonal IgG anti-human primary antibodies or 1:100 dilution PDZ-RhoGEF (N-14 sc-46234), 1:1000 dilution of MSN (C-15 sc 6410), or 1:1000 dilution of p-MSN (Thr 558 sc 12895) goat polyclonal IgG anti-human primary antibodies (Santa Cruz Biotechnology, Germany), or with a 1:5000–15,000 dilution of β-actin (ACTB) mouse monoclonal antibody clone number AC-15 (Sigma-Aldrich, Ireland), diluted in PBS containing 3% bovine serum albumin (w/v) or 5% low-fat milk powder (w/v) (Dawn Dairies, Ireland), 0.03% Tween 20 (v/v) and 0.1% NaN_3 _(v/v). Blots were then washed three times with PBS-0.03%T for 10 minutes each and incubated in a 1:4,000 dilution of a rabbit anti-goat horseradish peroxidase-conjugated antibody (DakoCytomation Ltd, Cambridgeshire, UK) or 1:1000 dilution anti-rabbit secondary (Pierce Technology, USA) or 1:4000 swine anti-rabbit IgG horseradish peroxidase-conjugated antibody (P-0217 DakoCytomation Ltd, Cambridgeshire, UK) or in 1:4000 dilution of a goat anti-mouse horseradish peroxidase-conjugated antibody (sc2005 Santa Cruz Biotechnology, Germany) or 1:1000 anti-mouse secondary (Pierce Technology, USA) in 1X PBS, 3%BSA (w/v), 0.05% Tween 20 (v/v) or in 1X PBS, 5% low-fat milk powder (w/v) (Dawn Dairies, Ireland) with 0.05% Tween 20 for 1 hour at room temperature. Blots were then washed with PBS-0.05%T. Bound secondary antibody was detected using the West-Pico chemiluminescent detection kit as per the manufacturer's protocol (Pierce Technology, USA) or Immobilon Western chemiluminescent HRP substrate (Millipore, USA). The membranes were scanned with the fluorescence imager (FluorchemTM 8900, Alpha Innotech Corporation, San Leandro, California, USA) and AlphaEaseFC software was used to detect the signal, the image was processed and protein expression levels where possible, were determined by densitometric analysis compared to corresponding levels of the housekeeping protein, β-Actin (ACTB). Statistical analysis of the densitometric data (one way ANOVA and Tukey's post hoc analysis) and graph construction were performed using GraphPad Prism version 4 (GraphPad Software Inc., USA).

### Immunofluorescence microscopy

Primary myometrial cells (to passage 8) were cultivated on LabTekII 8 well chamber slides (Nalge Nunc Int., Naperville, IL, USA) overnight. The samples were fixed in 4% paraformaldehyde (w/v) for 30 minutes at room temperature and blocked in 1X PBS, 0.01% Triton X-100 (v/v) and 5% serum (v/v) (donkey or goat). Cells were subsequently incubated with primary antibody in blocking solution, either a 1:50 dilution of MSN (C-15 sc 6410) goat polyclonal IgG anti-human primary antibody, p-MSN (Thr 558 sc12895) goat polyclonal IgG anti-human primary antibody or RHOGDI (ARHGDIA) (A20 sc360) rabbit polyclonal IgG anti-human primary antibody (Santa Cruz Biotechnology, Germany) in PBS/1%BSA overnight at 4°C. Samples were rinsed in 1XPBS 3 times and incubated with Alexa Fluor 488 donkey anti-goat IgG (A11055) or Alexa Fluor 488 goat anti-rabbit IgG (A11070) (Molecular Probes, Eugene, OR, USA) for 1 hour at room temperature and then rinsed in PBS. Control cells were incubated with the secondary antibody alone. After washing the coverslips were mounted on glass slides with Vectashield mounting medium with DAPI (Vector Laboratories, Burlingame CA, USA). Fluorescent images were obtained using the Laser Scanning Microscope LSM 510 (confocal microscope) (Carl Zeiss AG, Strasse 22, Oberkocken, Germany) and the DP70 fluorescence microscope (Olympus, Tokyo, Japan).

## Results

### RHOGTPase regulator mRNA expression in human myometrium

#### Semi-quantitative RT-PCR

**Figure 1:**
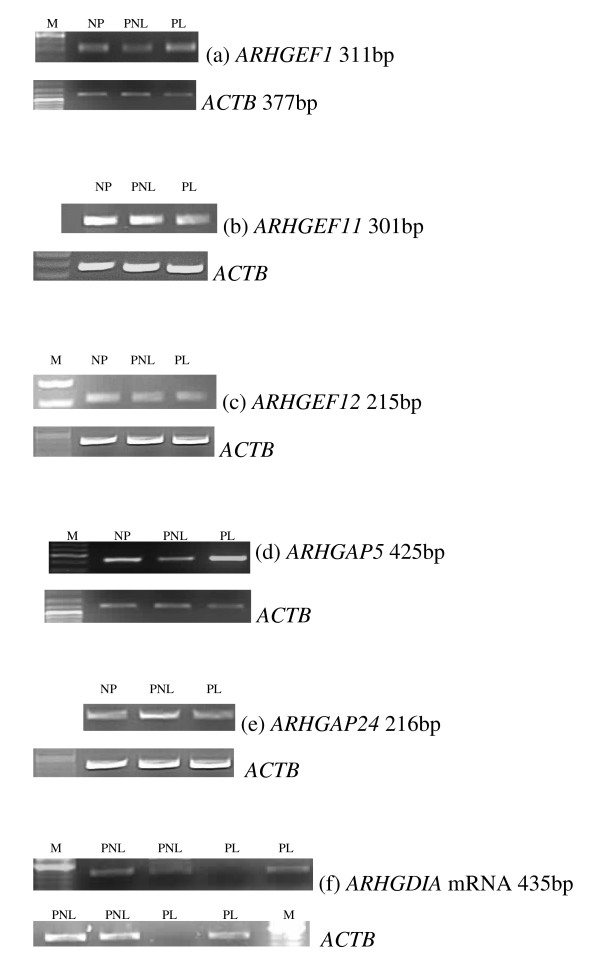
Representative RT-PCR gel pictures of (a) *ARHGEF1*, (b) *ARHGEF11*, (c) *ARHGEF12*, (d) *ARHGAP5*, (e) *ARHGAP24 *and (f) ARHGDIA mRNA expression in non-pregnant (NP), pregnant non-labouring (PNL) and pregnant labouring (PL) human myometrium. Corresponding *ACTB *expression for each gene is presented underneath each gel. The DNA marker 100 bp ladder (Promega, UK) is indicated (M) and sizes of PCR products presented.

RT-PCR analysis using DNA-free™ treated RNA demonstrated expression of *ARHGEF1*, *ARHGEF11*, *ARHGEF12*, *ARHGAP5*, *ARHGAP24 *and *ARHGDIA *mRNA in human non-pregnant (NP), pregnant non-labouring (PNL) and labouring (PL) myometrium (Figure 1a–f). The absence of transcripts in reverse transcriptase negative reactions (RT-) confirmed that all products were RNA derived and not generated from contaminating genomic DNA.

#### Real-time fluorescence RT-PCR

**Figure 2:**
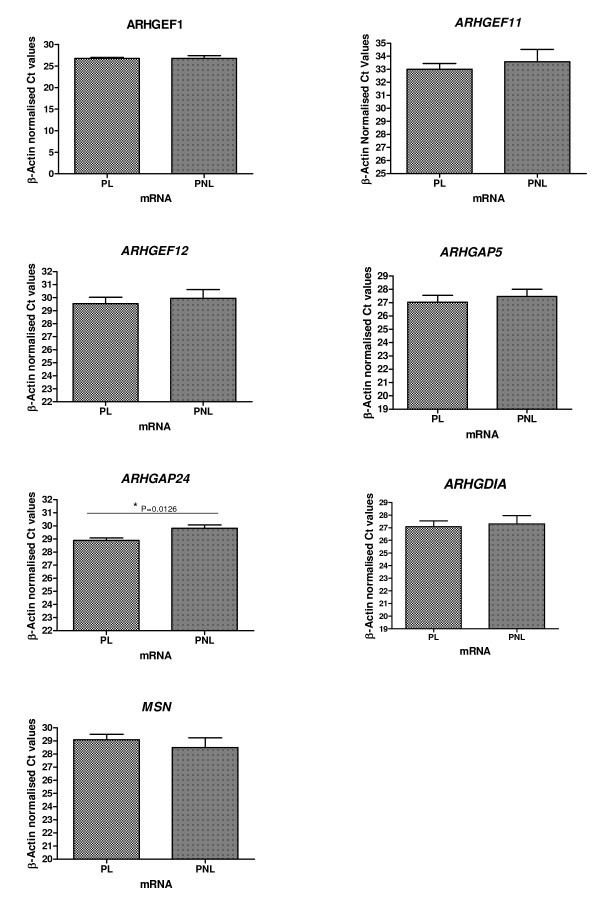
Graphical representations of real-time fluorescence RT-PCR results of β-Actin normalised Ct values plotted against myometrial pregnancy state (PL *n *= 7 and PNL *n *= 7) for each of the genes ± SEM (indicated by the error bars):*ARHGEF1*, *ARHGEF11*, *ARHGEF12*, *ARHGAP5*, *ARHGAP24*, *ARHGDIA *and *MSN*. Lower PL Ct values correspond to increased levels of mRNA expression for each gene at PL, in comparison to PNL.

**Figure 3:**
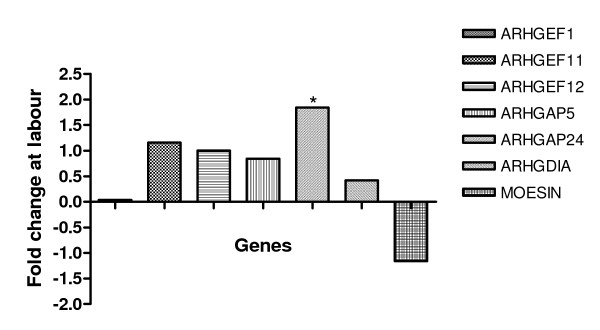
A summary of the fold changes for each gene at labour (*n *= 7) in comparison to the non-labouring state (*n *= 7) in human myometrium, normalised to β-Actin, from real-time fluorescence RT-PCR analyses. Fold change at labour is plotted against gene name: *ARHGEF1*, *ARHGEF11*, *ARHGEF12*, *ARHGAP5*, *ARHGAP24*, *ARHGDIA *and *MSN*, respectively. An asterisk indicates *P *< 0.05.

Relative quantitative expression analysis was then performed on the seven genes *ARHGEF1*, *ARHGEF11*, *ARHGEF12*, *ARHGAP5*, *ARHGAP24*, *ARHGDIA *and *MSN *by real-time RT-PCR. In order to minimise any undue experimental error from sources such as pipetting inaccuracies, analysis of each gene was performed in triplicate. RT-PCR product specificity was confirmed using melting curve analysis. Amplification curve crossing points were determined for each gene generated within the initial phase of exponential amplification, per 0.5 μg total RNA in the tissues studied. The mean Ct values for each transcript normalised to β-Actin (per 0.5 μg total RNA), were then averaged and values determined for both labouring (PL, *n *= 7) and non-labouring myometrium (PNL, *n *= 7). The mean β-Actin normalised Ct values for PL and PNL ± SEM, respectively for the 7 genes were: *ARHGEF1 *26.77 ± 0.23, 26.79 ± 0.6; *ARHGEF11 *33 ± 0.44, 33.58 ± 0.94, *ARHGEF12 *29.55 ± 0.48, 29.95 ± 0.67, *ARHGAP5 *27 ± 0.51, 27.46 ± 0.54, *ARHGAP24 *28.9 ± 0.19, 29.82 ± 0.25, *ARHGDIA *27.08 ± 0.46, 27.29 ± 0.67 and *MSN *29.09 ± 0.41, 28.5 ± 0.75 which is graphically represented in Figure 2. Relative fold changes were then calculated using the difference in the Ct values (x) between the PL and PNL myometrium for each transcript, Relative fold change = 2^x^. *ARHGAP24 *showed the greatest fold change increase at labour, by real-time RT-PCR which was significant (1.84-fold increase *P *= 0.0126) in comparison to the non-labouring at-term myometrium. *ARHGEF11*, *ARHGEF12*, *ARHGAP5*, *ARHGDIA *and *ARHGEF1 *showed slight increases in mRNA expression at labour, none of which were significant. A slight decrease in *MSN *expression at labour was also observed, which was not significant. A summary of the fold changes observed using real-time fluorescence RT-PCR is presented in Figure 3.

### RHOGTPase regulator expression in myometrial smooth muscle cells

**Figure 4:**
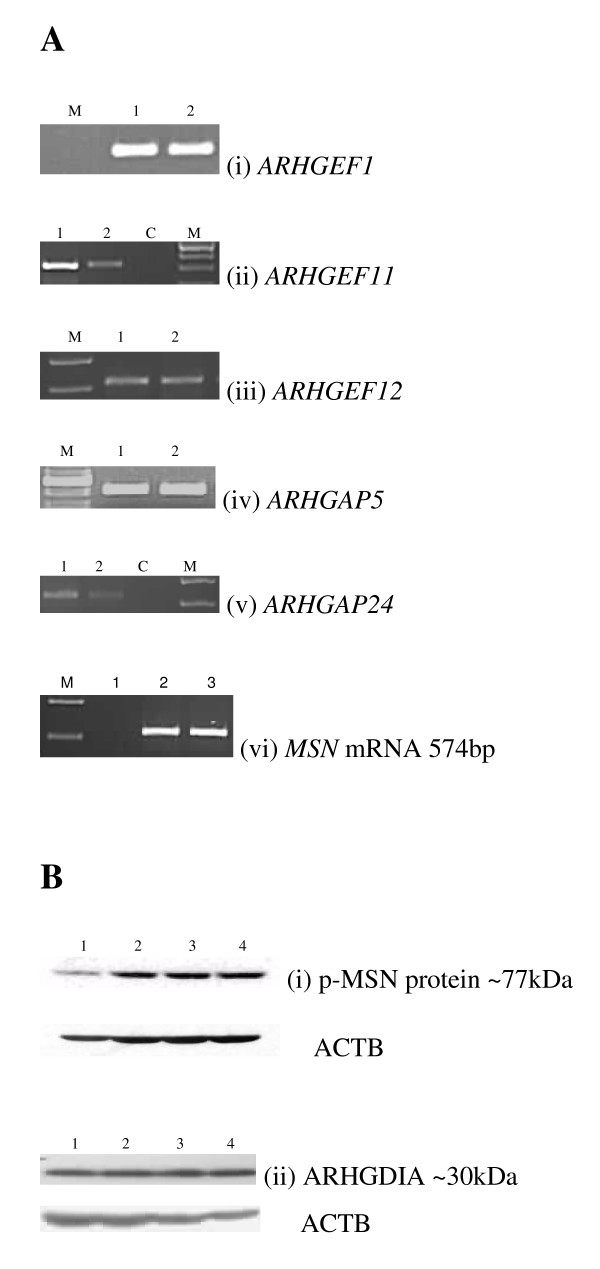
a. Representative RT-PCR gel pictures of (i) *ARHGEF1 *(ii) *ARHGEF11 *(iii) *ARHGEF12 *(iv) *ARHGAP5 *(v) *ARHGAP24 *and (vi) *MSN *mRNA expression in human myometrial cells (lanes 1 and 2 of each gel and lanes 2–3 for MSN). The PCR negative control is indicated (C). The DNA marker 100 bp ladder (Promega, UK) is indicated (M). b. Representative western blots of (i) p-MSN and (ii) ARHGDIA protein expression in human myometrial cells (lanes 1–4 of each gel). The corresponding β-Actin (ACTB) western blot is presented underneath the appropriate blot. Molecular weights are indicated in kDa.

RT-PCR analysis demonstrated expression of *ARHGEF1*, *ARHGEF11*, *ARHGEF12, ARHGAP5*, *ARHGAP24 *and *MSN *mRNA in human myometrial smooth muscle cells, that were sub-passaged from primary cells in culture (Figure 4a(i–vi)).

Western Blot analysis demonstrated expression of pMSN and ARHGDIA proteins in the human myometrial smooth muscle cells (Figure 4b(i–ii)).

### RHOGTPase regulator protein expression in human myometrium

#### RHOGEF, RHOGAP and RHOGDI protein expression in human myometrium

**Figure 5:**
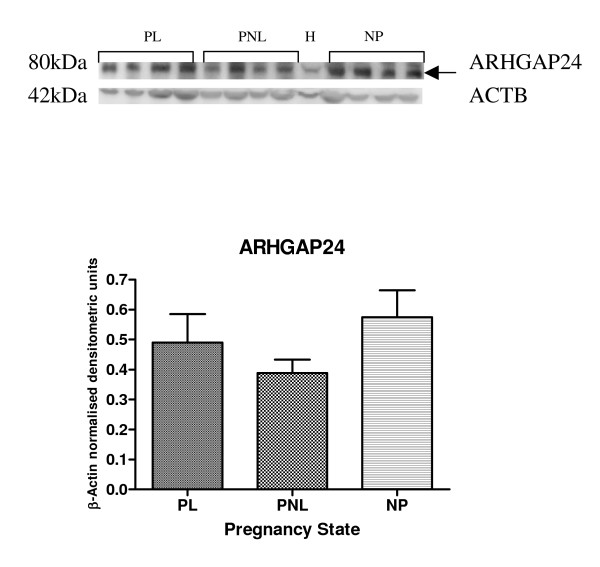
Representative Western blot of ARHGAP24 expression in samples of PL (*n *= 4), PNL (*n *= 4) and NP (*n *= 4) human myometrium, and HeLa cells (H). The corresponding ACTB protein expression is presented underneath. Protein bands of interest are indicated with arrows and molecular weights are indicated in kDa. Quantitative densitometric analysis of the western blot is presented, with β-Actin normalised densitometric units for each protein plotted against pregnancy state ± SEM (indicated with error bars).

**Figure 6:**
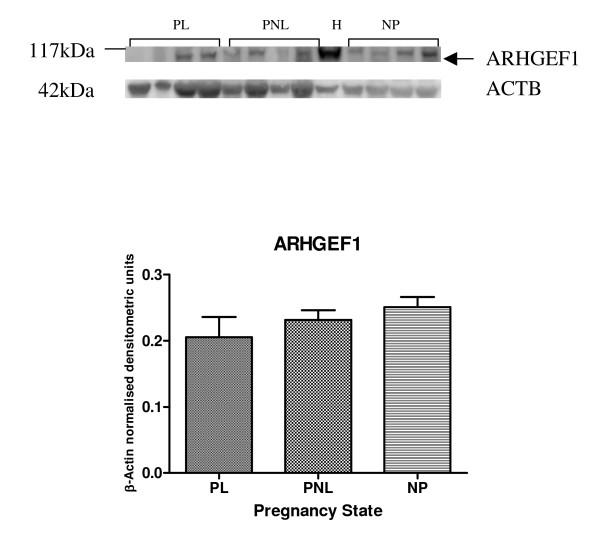
Representative Western blot of ARHGEF1 expression in samples of PL (*n *= 4), PNL (*n *= 4) and NP (*n *= 4) human myometrium, and HeLa cells (H). The corresponding ACTB protein expression is presented underneath. Protein bands of interest are indicated with arrows and molecular weights are indicated in kDa. Quantitative densitometric analysis of the western blot is presented, with β-Actin normalised densitometric units for each protein plotted against pregnancy state ± SEM (indicated with error bars).

**Figure 7:**
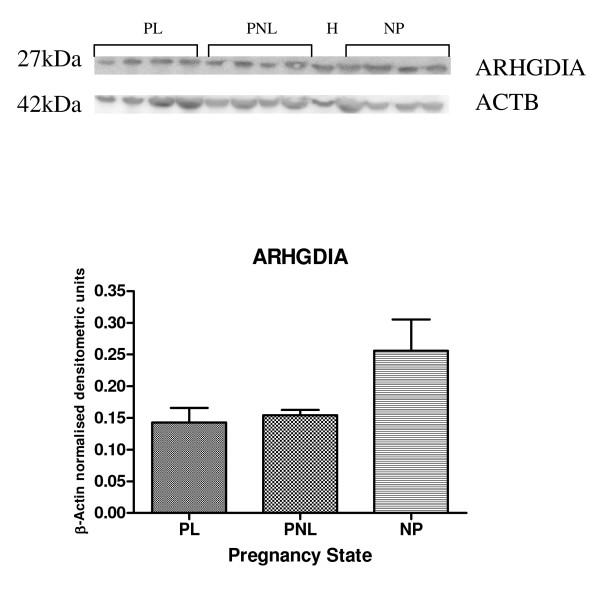
Representative Western blot of ARHGDIA expression in samples of PL (*n *= 4), PNL (*n *= 4), NP (*n *= 4) human myometrium, and HeLa cells (H). The corresponding ACTB protein expression is presented. Protein bands of interest are indicated with arrows and molecular weights are indicated in kDa. Quantitative densitometric analysis of the western blot is presented, with β-Actin normalised densitometric units for each protein plotted against pregnancy state ± SEM (indicated with error bars).

**Figure 8:**
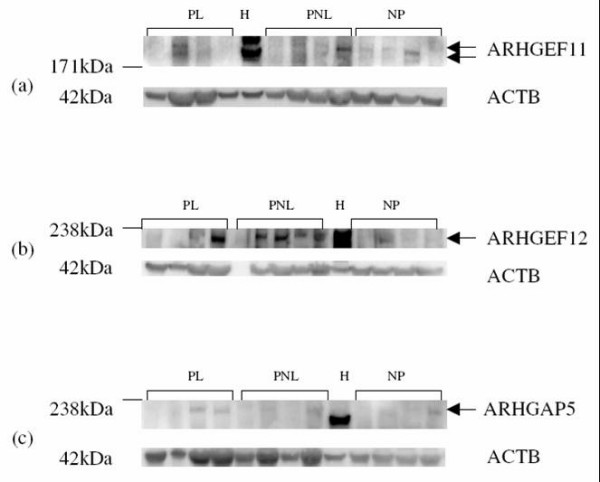
Representative Western blots of (a) ARHGEF11 (b) ARHGEF12 (c) ARHGAP5 expression in samples each of pregnant labouring (PL, *n *= 4) pregnant non-labouring (PNL, *n *= 4) and non-pregnant (NP, *n *= 4) human myometrium, and HeLa (H) cells. The corresponding ACTB protein expression is also presented. Protein bands of interest are indicated with arrows and molecular weights are indicated in kDa.

Protein expression of ARHGEF1, ARHGEF11, ARHGEF12, ARHGAP24 and ARHGDIA by western blotting was demonstrated in human myometrium (Figures 5, 6, 7 and 8). Bands of approximately the correct size of 115, 171, 230, 190, 80 and 27 kDa were obtained for ARHGEF1, ARHGEF11, ARHGEF12, ARHGAP5, ARHGAP24 and ARHGDIA respectively, in the myometrium. ARHGAP24 expression was previously demonstrated in HeLa cells [20] and these were used as a positive control for the western blot experiments in human myometrium. HeLa cell lysate was also used in the other western blots, with expression of ARHGEF1, ARHGEF11, ARHGEF12, ARHGAP5, ARHGAP24 and ARHGDIA protein reported in this cancer cell line. The mean β-Actin normalised densitometric units ± SEM for ARHGAP24 were PL (*n *= 4) 0.4902 ± 0.095, PNL (*n *= 4) 0.389 ± 0.044 and NP (*n *= 4) 0.5751 ± 0.089 (Figure 5). From the densitometric analyses therefore, there was a decrease in ARHGAP24 protein expression at PNL in comparison to NP and there was an increase in protein expression at PL in comparison to PNL.

There was no significant difference however in ARHGEF1 expression in myometrial NP, PNL or PL after Western blot densitometric analysis (Figure 6). The averaged β-Actin normalised densitometric units ± SEM for ARHGEF1 were PL (*n *= 4) 0.2055 ± 0.0303, PNL (*n *= 4) 0.2315 ± 0.015 and NP (*n *= 4) 0.251 ± 0.015 (Figure 6).

The mean β-Actin normalised densitometric units ± SEM for ARHGDIA were PL (*n *= 4) 0.142 ± 0.023, PNL (*n *= 4) 0.1541 ± 0.008 and NP (*n *= 4) 0.2561 ± 0.049 (Figure 7). From the densitometric data there was a decrease in ARHGDIA expression at PNL and PL in comparison to NP, and where both PNL and PL expression was similar. None of these results however, were significant.

The expression of ARHGEF11, ARHGEF 12 and ARHGAP5 protein was detected however the western blots were not suitable to perform densitometric analysis as expression was weak in some cases with some background on the blots (Figure 8a,b and 8c). Also, the expression of ARHGEF1, 11, 12 and ARHGAP5 was much weaker in the myometrium in comparison to that in the HeLa cells. Two protein bands were visible for ARHGEF11 both in the HeLa cells and in the myometrial tissue samples (Figure 8a). The doublet in the HeLa cells ran higher than those in the myometrial samples possibly due to differences in posttranslational modification or perhaps due to differences associated with the cancer cell line. The lower band in the myometrial samples corresponds most closely to the expected size of 167–171 kDa for human ARHGEF1. ARHGEF12 protein expression was also observed with a band of the expected size of 220–230 kDa with an observed increase in expression in PNL and PL in comparison to NP (Figure 8b).

ARHGAP5 expression was also detected in all the myometrial biopsies examined, with a difference in size, with the HeLa protein band being shifted down the gel, possibly due to alternative posttranslational modifications or a truncated ARHGAP5 version in the cancer cell line (Figure 8c). The band in the myometrial samples appears to be closer in size to that predicted (190 kDa) for human ARHGAP5.

#### MSN protein expression in human myometrium

**Figure 9:**
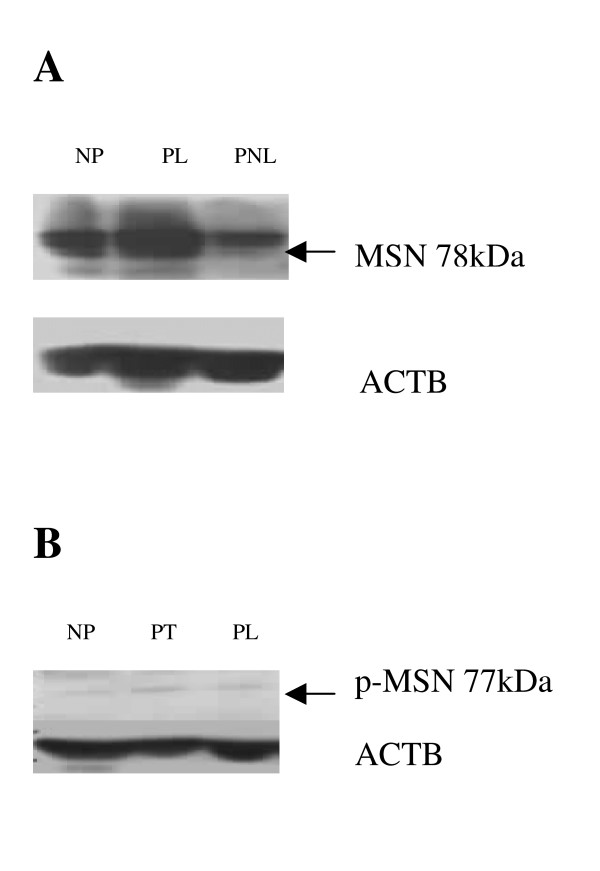
(a) Representative western blot of MSN protein expression (indicated with an arrow) in non-pregnant (NP), pregnant non-labouring (PNL) and pregnant labouring (PL) human myometrium. The corresponding ACTB expression is also presented. (b) Representative western blot of p-MSN protein expression (indicated with an arrow) in non-pregnant (NP), pregnant non-labouring (PT) and pregnant labouring (PL) human myometrium. Corresponding ACTB expression is presented underneath the blot.

**Figure 10:**
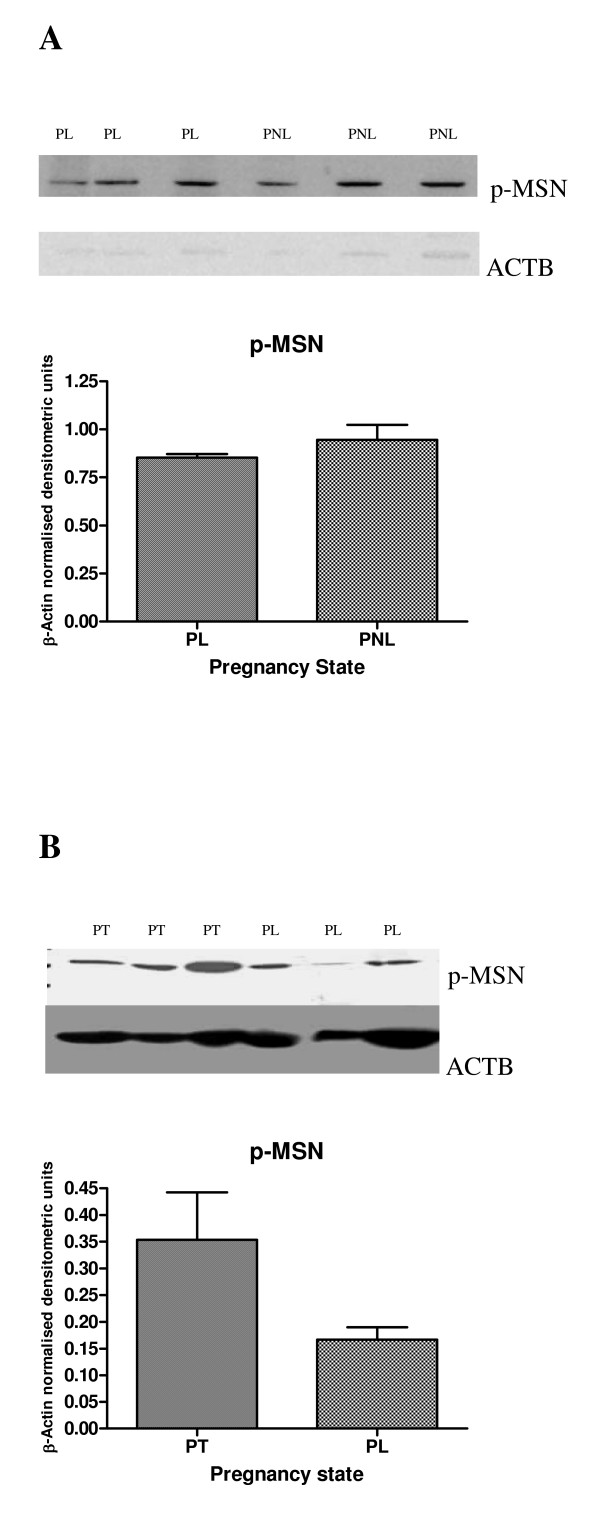
(a) Representative Western blot analysis of p-MSN expression with corresponding ACTB blot, in PL (*n *= 3) and PNL (*n *= 3) human myometrium. Densitometric analysis of the above p-MSN protein expression normalised to ACTB is also presented, with normalised densitometric units plotted against pregnancy state. (b) Representative Western blot analysis of p-MSN protein expression with corresponding ACTB blot, in PT (*n *= 3) and PL (*n *= 3) human myometrium. Densitometric analysis of the above p-MSN protein expression normalised to ACTB is also presented, with normalised densitometric units plotted against pregnancy state.

Western blotting analysis demonstrated MSN protein expression (78 kDa band) in human NP, PL and PNL myometrium (Figure 9a). Phosphorylated moesin or active moesin, p-MSN, protein expression was also observed in NP, PNL, PL and in PT myometrium (Figure 9b and Figure 10a and 10b). No significant difference in p-MSN protein band intensity was evident between the myometrial biopsies (PL v PNL) or (PT v PL), after densitometric analysis (Figure 10a and 10b). The mean β-Actin normalised densitometric units ± SEM for p-MSN (PL v PNL) (Figure 10a) were PL (*n *= 3) 0.853 ± 0.017, PNL (*n *= 3) 0.947 ± 0.076 and for p-MSN (PT v PL) densitometry (Figure 5d) PT (*n *= 3) 0.353 ± 0.089, PL (*n *= 3) 0.167 ± 0.023 (Figure 10b). Summarising the densitometric data there was a decrease in p-MSN band intensity at PL in comparison to PNL, though this was not significant (Figure 10a). Also there was a slight increase in p-MSN band intensity in the PT myometrium, in comparison to the PL tissue, though this also was not significant (Figure 10b).

### ARHGDIA and MSN immunolocalisation in human myometrial smooth muscle cells

**Figure 11:**
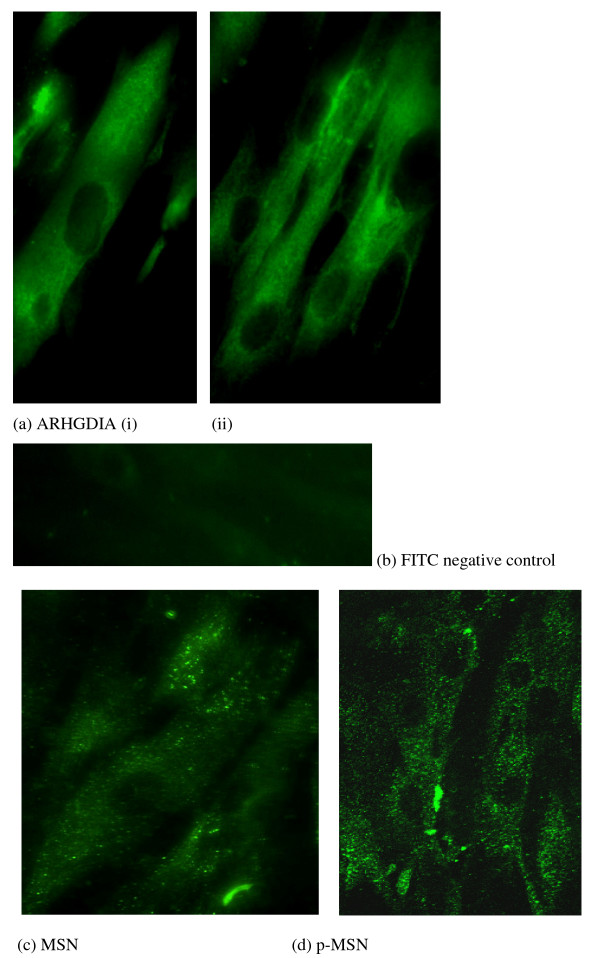
**Immunofluorescence confocal microscopy**: (a(i-ii)) ARHGDIA and (c) MSN and (d) p-MSN confocal microscopy immunolocalisation in human primary myometrial smooth muscle cells. A FITC negative control is presented (b) and the original magnification was ×40.

Immunolabelling confocal microscopy localised ARHGDIA protein to the cytoplasm of human myometrial smooth muscle cells (Figure 11a (i) and (ii)). MSN and pMSN immunofluorescence studies localised the proteins to vesicle structures in the cytoplasm of the myometrial smooth muscle cells (Figure 11c and 11d).

## Discussion

It is now established that RHOA, B and D and RND molecules of the RHOGTPase subfamily of RAS proteins are involved in the regulation of myometrial function during pregnancy and labour. Regulation of these myometrial GTPases is therefore essential, at this critical time.

We have determined that some RHOGTPase regulators, ARHGEF1, ARHGEF11, ARHGEF12, ARHGAP5, ARHGAP24, ARHGDIA and MSN are expressed in human myometrium during pregnancy, labour and in the non-pregnant state. Their expression was also detected in the smooth muscle cells of this tissue. All three RHOGEFs, ARHGEF1, ARHGEF11, ARHGEF12 were previously reported in vascular smooth muscle and exhibited very selective activation of RHOA [13,34,35]. ARHGEF1 mRNA or protein expression did not significantly alter in the myometrium at term pregnancy, at labour or in the non-pregnant state.

ARHGEF11 has the ability to induce calcium sensitisation in rabbit pulmonary artery smooth muscle [14]. Furthermore, ARHGEF11 myometrial mRNA expression was reported to be up-regulated after estradiol treatment of ovariectomised guinea pigs [15]. However ARHGEF11 mRNA expression did not vary in the pregnant human myometrium. Moreover, the expression of ARHGEF11 protein was weaker in all the myometrial samples analysed, in comparison to the HeLa cells. ARHGEF12 mRNA and protein expression was detected in human myometrium and on the myometrial smooth muscle cells. However, no significant difference in gene expression was observed between the myometrial samples at the different time-points.

The expression of two RhoGTPases, ARHGAP5 and ARHGAP24 was observed in human myometrium and on myometrial smooth muscle cells, with a significant 1.84-fold increase in *ARHGAP24 *mRNA and a slight increase in protein expression at labour in comparison to the non-labouring state. In vascular smooth muscle cells it has been determined that ARHGAP24 is a key regulator of angiogenesis, where it displays GTPase activity to RHO [18]. It is also known that it acts as a GTPase activator for RAC by converting it to an inactive GDP bound state. It controls actin remodelling by inactivating RAC, downstream of Rho [19]. ARHGAP24 can also suppress RAC and CDC42 *in vitro*, and its over-expression induces cell rounding with disruption of actin stress fibres and formation of membrane ruffles, lamellipodia and filopodia [20]. The observed increase in ARHGAP24 expression at labour in the myometrium may result in the inhibition of RHOGTPases, perhaps RAC and CDC42 or others, perhaps resulting in the alteration of uterine smooth muscle contractility at labour.

ARHGAP5 or p190B-RhoGAP as it is also known, is ubiquitously expressed and it has previously been established that it displays specificity for RHOA, RAC and CDC42 [8,36]. Recruitment of it's isoform, p190-RHOGAP is associated with RHOA inactivation and inhibition of contractility in rat vascular smooth muscle cells [21]. ARHGAP5 myometrial mRNA expression was not significantly different at term pregnancy or at labour. ARHGAP5 protein expression was demonstrated in human myometrium and mRNA expression determined on myometrial smooth muscle cells.

RHOGDIs are also thought to play an important role in regulating the association of RHOGTPases with cell membrane compartments. This is based on their ability to mask the prenyl group of post-translationally modified GTPases, thereby inhibiting the interaction of these GTPases with membranes [9]. ARHGDIA can bind to RHOA and B, RAC1 and 2, and CDC42 and therefore result in their inhibition by binding to these RHOGTPases and maintain them in the cytosol [23]. We observed *ARHGDIA *mRNA and protein expression in human myometrium at term labour, at labour and in the non-pregnant state and also in human myometrial smooth muscle cells. A decrease in its protein expression at labour and at term pregnancy, in comparison to the non-pregnant state, suggests decreased RHOGDI expression may result in increased RHOGTPase activation observed during pregnancy and labour [4-6] by being unable to bind to the RHOGTPases in the cytosol. Immunofluorescent confocal microscopy localised ARHGDIA protein to the cytoplasm of myometrial smooth muscle cells. The decrease in expression of ARHGDIA at labour and at the end of pregnancy may result in the decrease of its inhibitory effect on RHOGTPases either RHOA or RHOB or other RHOGTPases, thus enabling them to be activated by RHOGEFs.

Although RHOGEFs have received much attention as key players in GTPase activation, there is also evidence to suggest that RHOGAPs and RHOGDIs may play a role in the activation of the RHOGTPase cycle and do not just deactivate or inhibit GTPases. For example, the interaction between RHOGDI and RHO can be inhibited by binding of RHOGDI to members of the ERM family, that serve as crosslinkers between actin filaments and the plasma membrane [27]. The modulation of ERM protein activity therefore may contribute to the activation of GTPases, presumably by sequestering RHOGDI [37].

The expression of the ERM, MSN was observed for the first time in this study in the human myometrium, and its expression demonstrated in human myometrial smooth muscle cells. Activated or phosphorylated MSN protein was also observed in the smooth muscle cells. This key protein can cross-link the cortical actin cytoskeleton to plasma membrane protein, regulating cell motility and adhesion and is also a RHO downstream effector. This molecule therefore, serves an important function in all cells, enabling cell adhesion and actin cytoskeletal movement of cells. Myometrial smooth muscle MSN may be activated during contraction and thus result in the activation of RHOGTPases through the displacement of RHO from RHOGDI.

RHOGTPases play many significant roles in the body and none more so than in the myometrium during pregnancy and labour. Regulation of these molecules is vital as any error in their control could have serious consequences, such as in preterm labour. The expression of the various RHOGEFs, RHOGAPs, a RHOGDI and MSN in the human myometrium and the increase in ARHGAP24 expression at labour suggests a complex RHO regulatory mechanism exists in this tissue which may play a role in the control of myometrial contractility. Further investigation however, is necessary to fully elucidate the biochemical pathways involved.

## Conclusion

This study demonstrated the expression of ARHGEF1, ARHGEF11, ARHGEF12, ARHGAP5, ARHGAP24, ARHGDIA and MSN in human myometrium, at term pregnancy, at labour, in the non-pregnant state and in myometrial smooth muscle cells. It was also determined that *ARHGAP24 *mRNA expression significantly increased at labour. These data demonstrate the expression of RHOGTPase regulatory molecules in the myometrium and suggest some may play a role in the control of contractility in this tissue at labour.

## Competing interests

The author(s) declare that they have no competing interests.

## Authors' contributions

MOB conceived the idea and design of the project, acquired the data, drafted the manuscript, performed semi-quantitative RT-PCR, fluorescence real-time RT-PCR, tissue westerns, cellular westerns and immunofluorescence microscopy. DF performed tissue westerns and BM performed semi-quantitative RT-PCR. JJM provided the myometrial tissue samples, read and edited the final document. TJS acquired funding, supervised the research group, read and edited the final document. All authors read and approved the final document.
